# Mass Spectrometric Characteristics of Prenylated Indole Derivatives from Marine-Derived *Penicillium* sp. NH-SL

**DOI:** 10.3390/md15030086

**Published:** 2017-03-22

**Authors:** Hui Ding, Wanjing Ding, Zhongjun Ma

**Affiliations:** Institute of Marine Biology, Ocean College, Zhejiang University, Zhoushan Campus, No. 1 Zheda Road, Zhoushan 316021, China; snnu_dh@163.com (H.D.); dwj1988555@126.com (W.D.)

**Keywords:** prenylated indole alkaloids, *Penicillium* sp. NH-SL, LC-MS/MS analysis, cytotoxic activity, mass spectrometric characteristics

## Abstract

Two prenylated indole alkaloids were isolated from the ethyl acetate extracts of a marine-derived fungus *Penicillium* sp. NH-SL and one of them exhibited potent cytotoxic activity against mouse hepa 1c1c7 cells. In order to detect other bioactive analogs, we used liquid chromatogram tandem mass spectrometry (LC-MS/MS) to analyze the mass spectrometric characteristics of the isolated compounds as well as the crude extracts. As a result, three other analogs were detected, and their structures were deduced according to the similar fragmentation patterns. This is the first systematic report on the mass spectrometric characteristics of prenylated indole derivatives.

## 1. Introduction

Natural products are important sources of drugs and drug leads [1,2]. In recent years, numerous biologically active natural products have been isolated from microbes collected from different environments such as plants, animals, soils, and sediments [3]. Marine microbes have been proven to possess a unique metabolic mechanism and an adaptive mechanism due to their special living environment, which is quite different from a terrestrial environment specialized in high pressure, high salt, low temperature, and a lack of nutrition [4]. As an essential part of marine microbes, marine-derived fungi have proven to be a major source of marine natural products due to their complex genetic background and chemodiversity [5,6]. Among them, the genus *Penicillium* has received great attention among all marine-derived fungi, which account for 22% of all natural products of marine fungal origin [7]. Their various secondary metabolites usually have uncommon structures and potent bioactivities that might possess substantial pharmaceutical values [8].

Prenylated indole alkaloids are widely distributed in marine organisms, especially in the genera *Penicillium* [9]. These compounds carry a prenyl moiety at Position C-3 of the indoline ring and a five-membered ring system between the indoline and the diketopiperazine ring in their structures. The biogenetic origin of indole or indoline ring is tryptophan, which is a key precursor from primary metabolism [10]. The diketopiperazine structure is formed by cyclization of tryptophan and a second amino acid, which usually appeared as proline, alanine, phenylalanine, or valine [11]. Prenylated indole alkaloids often carry biological and pharmacological activities distinct from their non-prenylated aromatic precursors [9]. However, very few reports concentrate on the rapid detection and identification of prenylated indole alkaloids in microbial extracts.

Although the most efficient method for complete structure elucidation of natural products is nuclear magnetic resonance spectroscopy (NMR), this method requires relatively large amounts of purified sample. For the rapid identification of minor compounds in complex mixtures, tandem mass spectrometry is an effective method. This technique has been used as an analytical tool for the characterization of specific compounds in complex mixtures such as extracts from microorganisms [12,13], marine invertebrates [14], algae [15], and plants [16,17,18]. This research describes the mass spectrometric characteristics of prenylated indole alkaloids using LC-MS/MS with the aim of developing a methodology for the rapid identification of prenylated indole alkaloids in microbial extracts.

## 2. Results and Discussion

### 2.1. Identification of Prenylated Indole Alkaloids

In our course of searching for bioactive prenylated indole alkaloids from marine microbes, a strain of *Penicillium* sp. NH-SL isolated from the deep-sea sediment samples of the South China Sea attracted our attention. The ethyl acetate extracts of the strain’s liquid fermentation culture showed an inhibition of 76% against mouse hepa 1c1c7 cells at a concentration of 20 µg/mL. Using silica gel column chromatography and high performance liquid chromatography (HPLC), two known prenylated indole alkaloids were isolated from this strain. 

Compound **1** was obtained as a white amorphous powder. Inspection of the NMR data (Table S1, Supplementary Materials) showed the same signals as those of brevicompanine B reported in the literature [19], so Compound **1** was identified as brevicompanine B. Compound **2** was also obtained as a white amorphous powder. The NMR data (Table S1, Supplementary Materials) were the same as those of verrucofortine reported in the literature [20], so Compound **2** was identified as verrucofortine.

**Figure 1:**
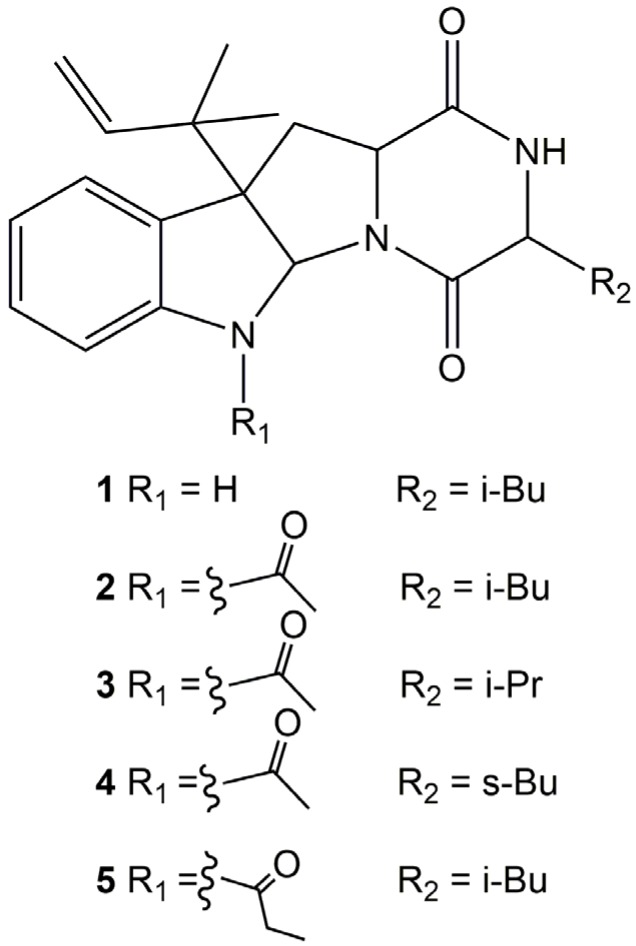
Structures of Compounds **1**–**5**.

According to the literature report, Compound **2** showed an inhibitory effect on plant growth while its analogs rugulosuvines A and B had moderate cytotoxicity against L-929, K562, and Hela cells [21]. This encouraged us to find out other analogs of these alkaloids. Finally, three other analogs, **3**–**5** [22,23], were detected by LC-MS/MS and their structures were deduced as shown in Figure 1.

### 2.2. MS/MS Analysis of Compounds ***1***–***5***

**Figure 2:**
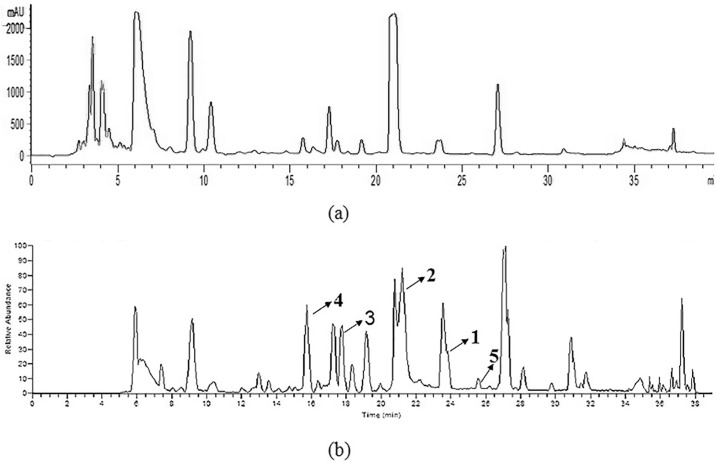
LC-MS/MS analysis of *Penicillium* sp. NH-SL’s fermentation extracts. (**a**) UV chromatogram at 250 nm; (**b**) TIC chromatogram.

**Figure 3:**
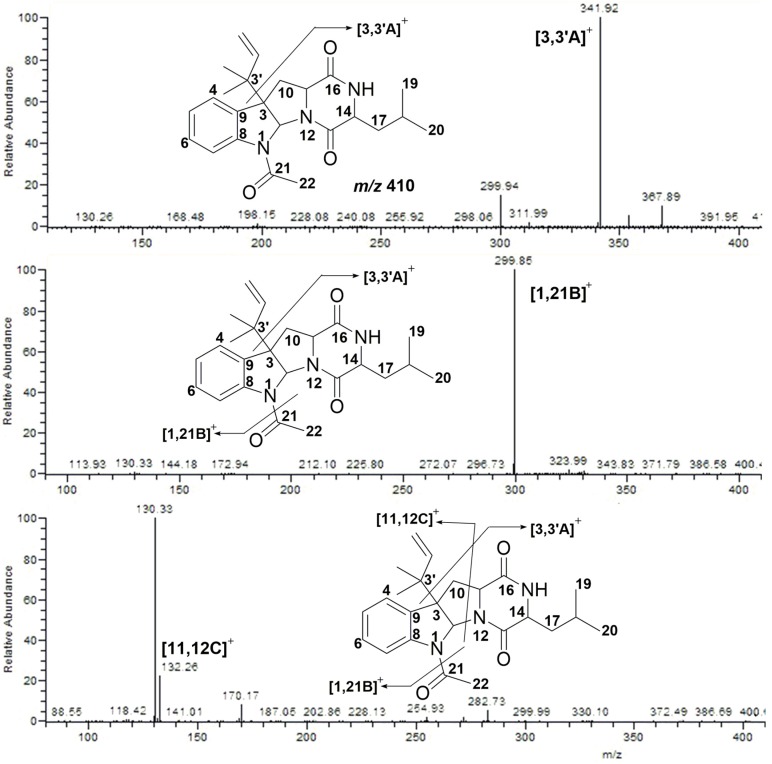
MS/MS spectra of Compound **2**.

The total prenylated indole derivatives were obtained from the ethyl acetate extracts of the strain’s fermentation broth. Figure 2 shows the UV and TIC chromatograms of the crude extracts. The mass spectra characteristics of Compound **2** were analyzed in order to find out other analogs (Figure 3). 

**Figure 4:**
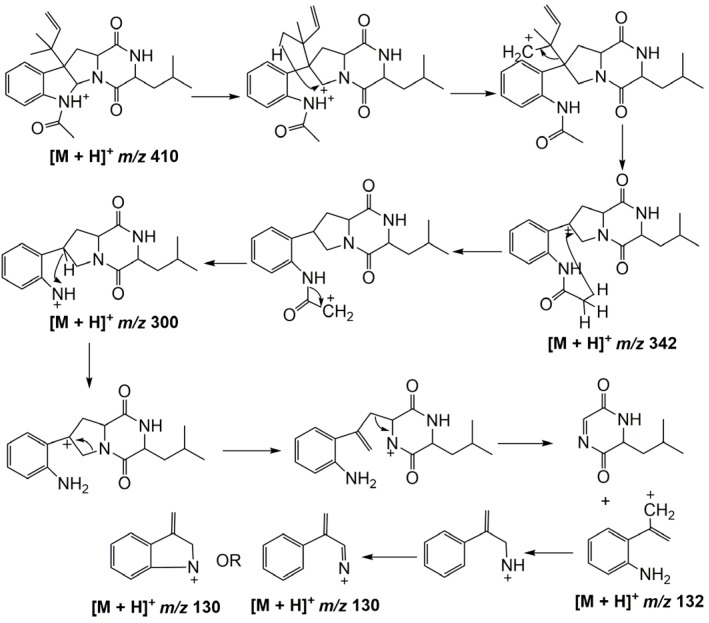
Cleavage pathway of Compound **2**.

Compound **2** exhibited [M + H]^+^ at *m/z* 410. It lost an isopentene group and fragmented into *m/z* 342 ([3,3′A]^+^). Then, an acyl group was lost and the compound further fragmented into *m/z* 300 ([1,21B]^+^). When it came to the fourth cleavage stage, two fragmentation products at *m/z* 132 ([11,12C]^+^) and *m/z* 130 were observed, respectively. The cleavage pathway is shown in Figure 4.

**Figure 5:**
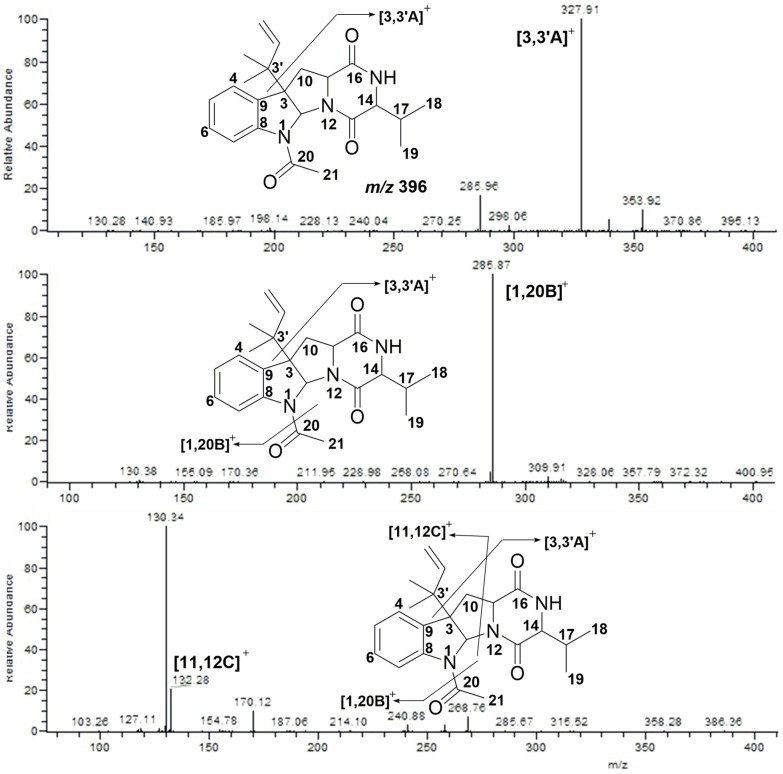
MS/MS spectra of Compound **3**.

According to the fragmentation patterns of Compound **2**, three other analogs were detected. Compound **3** exhibited [M + H]^+^ at *m/z* 396. It lost an isopentene group and fragmented into *m/z* 328 ([3,3′A]^+^). Successively, an acyl group was lost and the compound fragmented into *m/z* 286 ([1,20B]^+^). Further cleavage of [1,20B]^+^ yielded ion fragments *m/z* 132 ([11,12C]^+^) and *m/z* 130. The MS/MS spectra are shown in Figure 5.

**Figure 6:**
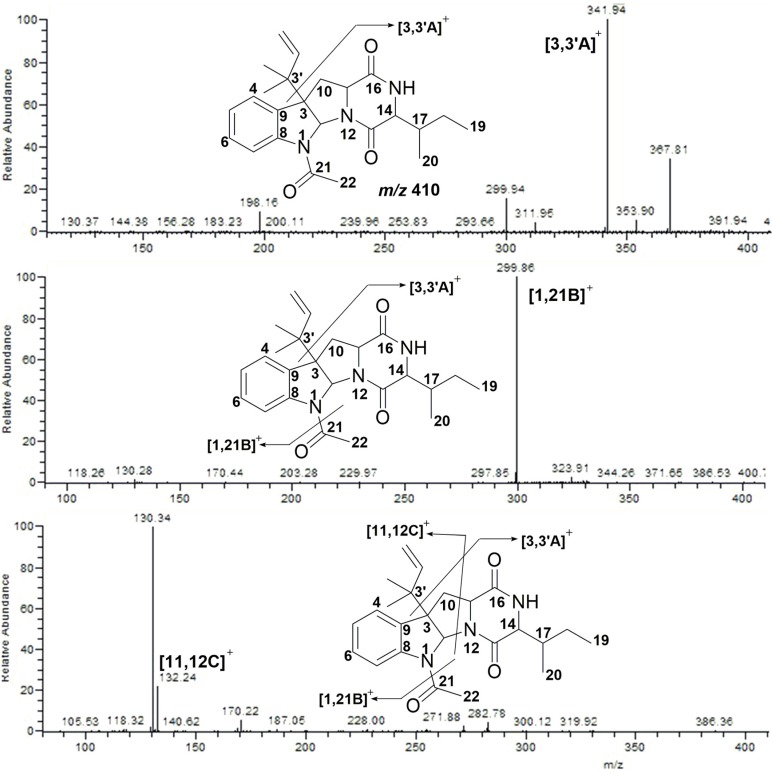
MS/MS spectra of Compound **4**.

Compound **4** displayed [M + H]^+^ at *m/z* 410, which indicated that Compound **2** and Compound **4** were isomerides. Further cleavage of Compound **4** yielded ion fragments *m/z* 342 ([3,3′A]^+^), *m/z* 300 ([1,21B]^+^), *m/z* 132 ([11,12C]^+^) and *m/z* 130, respectively, which were consistent with that of Compound **2** (Figure 6).

**Figure 7:**
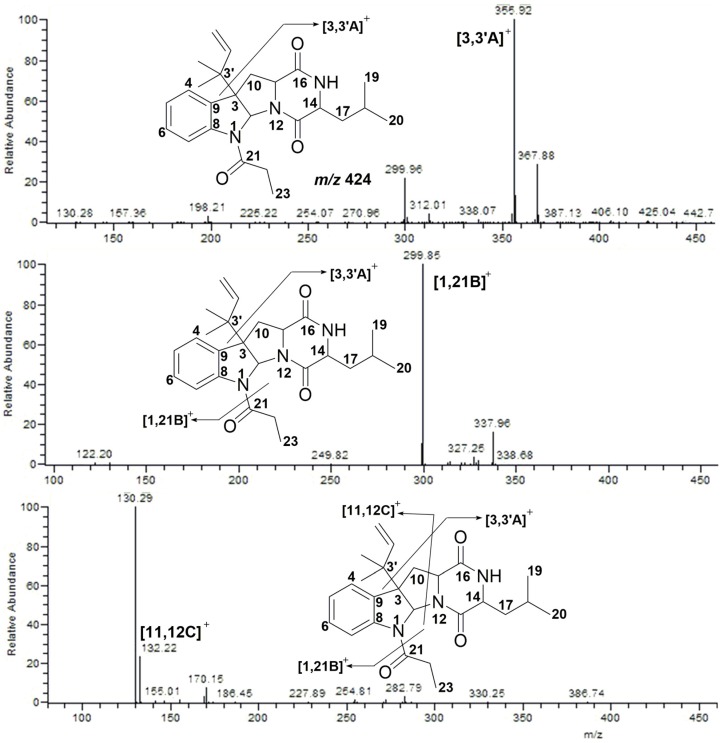
MS/MS spectra of Compound **5**.

Compound **5** displayed [M + H]^+^ at *m/z* 424. It lost an isopentene group and a C_3_H_5_O group, and fragmented into *m/z* 356 ([3,3′A]^+^) and *m/z* 300 ([1,21B]^+^), respectively. Finally, the ion fragment [1,21B]^+^ was cleaved into the typical ions *m/z* 132 ([11,12C]^+^) and *m/z* 130 (Figure 7).

To sum up, we found that the isopentene group at C-3 was usually cleaved from the skeleton and generated a loss of 68 u during the second cleavage stage. Then, a loss of the substitute group at Position N-1, which usually appeared as an acyl group, was observed during the third cleavage stage. When it came to the fourth cleavage stage, ion fragments *m/z* 132 as well as *m/z* 130 occurred, and these fragments were definitively identified as a character in the indole alkaloids’ cleavage pathway.

### 2.3. Cytotoxicity Assay

A cytotoxicity assay of Compounds **1** and **2** was performed using mouse hepa lclc7 cells. They showed no obvious activity when the testing concentration was 20 nM. However, when the testing concentration increased to 700 nM, Compound **2** exhibited a potent inhibition rate of 85%. The data of Compound **1** was not available at the same testing concentration due to its small quantity (Table 1). 

## 3. Experimental Section

### 3.1. Fungus Material

*Penicillium* sp. NH-SL was isolated using the standard agar plate dilution method from the deep-sea sediment samples of South China Sea and identified by TaKaRa Biotechnology Co., Ltd. (Dalian, China) by observing the morphological characteristics and analysis of the 26s rDNA regions (GenBank accession number: KY 378942.1). The strain was deposited in BeNa Culture Collection (BNCC 147224), Beijing, China. 

### 3.2. Growing Biomass and Crude Extracts Preparation

*Penicillium* sp. NH-SL was maintained on potato dextrose agar (PDA) medium at 28 °C for seven days. Spores of the strain were inoculated into 15 bottles of 500 mL Erlenmeyer flasks, each containing 250 mL of 2216E medium using a sterile inoculation loop and incubated at 28 °C on a rotary shaker at 180 rpm for three days. Large scale liquid fermentation (180 L) was carried out in 720 bottles of 500 mL Erlenmeyer flasks, each containing 250 mL of 2216E medium. Each flask was inoculated with 5.0 mL of culture medium and incubated at 28 °C for 12 days. After the large scale fermentation, all the culture broth was filtered through a four layer gauze in order to get rid of the biomass. Then, the fermentation broth was extracted with equal volume of ethyl acetate for twice to yield 8.0 g crude extracts. Ethyl acetate was purchased from Tianjin Damao Chemical Reagents Co., Ltd (Tianjin, China). Yeast extract and peptone used in the preparation process of 2216E culture medium were purchased from OXOID Ltd. (Basingstoke, UK). Deionized water was prepared using a Milli-Q system (Millipore, Bedford, MA, USA).

### 3.3. Fractions Isolation 

The crude extracts (8.0 g) was then subjected to silica gel column chromatography (5 × 60 cm, 120 g silica gel) and eluted with a gradient of dichloromethane/methanol (150:1, 100:1, 20:1, 5:1, 1:1, 0:1, *v*/*v*, each 1.2 L) to afford 10 fractions (Fractions A–J). Fraction E (200 mg) was separated by preparative HPLC directly using CH_3_OH·H_2_O as the mobile phase with a gradient program from 45% to 100% methanol subjected from 0 to 100 min (a flow rate of 10 mL/min) to afford Compounds **1** (1.0 mg, t_R_ = 60 min) and **2** (3.0 mg, t_R_ = 55 min). Structures of these two compounds were identified by NMR analysis (Table S1) and compared with literature data [19,20]. NMR spectra were recorded on Bruker Ultrashield 600 MHz spectrometer (Bruker Technologies Beijing Co., Ltd., Beijing, China) using DMSO-*d*_6_ as solvents with tetramethylsilane as the internal standard. Preparative HPLC using an Agilent-1200 system (Agilent Technologies China Co., Ltd., Shanghai, China) with a photodiode array detector and a Zorbax-C18 column (21.2 × 250 mm, 7 µm) were used to purify compounds isolated from silica gel column chromatography.

### 3.4. LC-MS/MS Analysis

To analyze, we used LC-MS/MS to detect whether there were other analogs of indole alkaloids in the crude extracts. The 20 mg ethyl acetate part of the crude extracts was suspended in methanol (1.0 mL). The solution was filtered through a 0.22 µm microporous membrane prior to LC-MS/MS analysis. All of the mass experiments were performed on a LCD Deca XP MAX mass spectrometer (ThermoFisher, Waltham, MA, USA), which was connected to an Agilent 1200 HPLC instrument (Agilent, Karlsruhe, Germany) via an ESI interface. Ultra-high purity helium was used as the collision gas and high-purity nitrogen as the nebulizing gas. The ionization source parameters were set up as follows: The ion spray voltage and fragmentation voltage were set up at 4.5 kV and 25 V, respectively. The sheath gas (N_2_) and auxiliary gas (N_2_) were set up at 50 units and 10 units, respectively. The capillary temperature was set up at 330 °C, and the collision energy for CID was adjusted to 35% of maximum. In the whole system, A (H_2_O) and B (CH_3_OH) were used as the mobile phase. A gradient program was applied according to the following method: 0–8 min, 55% B, 8.01–30 min, 60%–80% B, 30.01–40 min, 100% B. The injection volume was 6 µL with a flow rate of 0.6 mL/min. The column and sample temperature were maintained at 35 °C and 25 °C, respectively. All experiments were carried out in positive ionization mode.

### 3.5. Cytotoxicity Assay

The cytotoxicity of the crude extracts and Compounds **1** and **2** against mouse hepa lclc7 cells (obtained from ATCC) was determined by the MTT method. Cells were plated in a 96-well plate 24 h before treatment. After that, the test compounds were added, and the cells were incubated for an additional 48 h. The cytotoxicity of hepa lclc7 cells and the isolated compounds were determined by a crystal violet assay as previously described [24]. Experiments were carried out three times on separate occasions.

## 4. Conclusions

In conclusion, prenylated indole alkaloids were isolated from the secondary metabolites of *Penicillium* sp. NH-SL and they were systematically analyzed by LC-MS/MS for the first time. From the crude extracts, two identified prenylated indole alkaloids together with three deduced analogs were characterized based on their similar fragmentation patterns in mass spectra. All of them showed a loss of an isopentene group at Position C-3 and successively a loss of the substitute group at Position N-1 during the second and third cleavage stage, respectively. They finally generated two ion fragments *m/z* 132 and *m/z* 130, which were identified as characters in the indole alkaloids’ cleavage pathway. Among these compounds, verrucofortine exhibited a potent inhibition rate of 85% against mouse hepa lclc7 cells at the concentration of 700 nM. This research provided a valuable method for the rapid detection and identification of bioactive prenylated indole alkaloids in microbial extracts.

## Figures and Tables

**Table 1:** Cytotoxic activities of the crude extracts and Compounds **1** and **2** ^a^.

Compounds	Inhibition Rates against Hepa Lclc7 Cells		
	20 µg/mL	20 nM ^c^	700 nM ^c^
crude extracts	76%	**-**	-
**1**	-	<10%	-
**2**	-	<10%	85%
cisplatin ^b^	IC_50_ = 10.7 nM		

^a^ Results are expressed as means ± SD (*n* = 3). ^b^ Positive control substance. ^c^ 20 nM and 700 nM were the maximum concentrations that could be obtained after samples recycled for Compounds **1** and **2**, respectively.

## Supplementary Materials

The following are available online at www.mdpi.com/1660-3397/15/3/86/s1, Figure S1: ^1^H NMR spectrum of Compound **1** in DMSO-*d*_6_, Figure S2: ^13^C NMR spectrum of Compound **1** in DMSO-*d*_6_, Figure S3: ^1^H NMR spectrum of Compound **2** in DMSO-*d*_6_, Figure S4: ^13^C NMR spectrum of Compound **2** in DMSO-*d*_6_, Table S1: ^1^H and ^13^C NMR data of Compounds **1** and **2**.
