# Supplementary material for: Mass Spectrometric Characteristics of Prenylated Indole Derivatives from Marine-Derived Penicillium sp. NH-SL
Source: Mar Drugs. 2017 Mar 22;15(3):86. doi: 10.3390/md15030086 (PMC5367041; doi:10.3390/md15030086)
Supplement: Supplementary file 1 [file marinedrugs-15-00086-s001.pdf]

SupplementaryMaterials: Mass Spectrometric Characteristics of Prenylated Indole Derivatives from Marine-derived *Penicillium* sp. NH-SL

Hui Ding, Wanjing Ding and Zhongjun Ma*

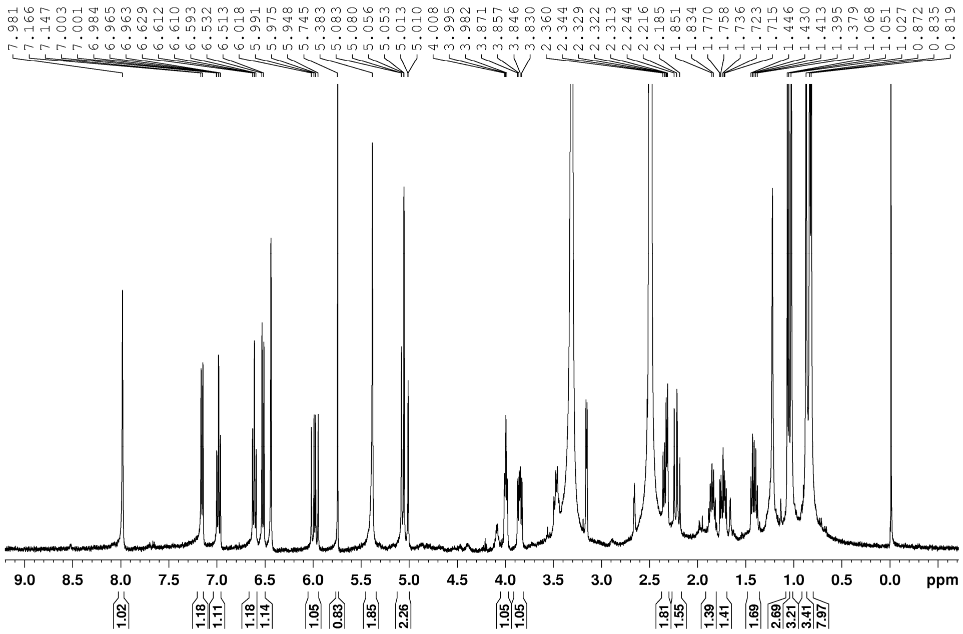

Figure S1. ^{1}H NMR spectrum of Compoud **1** in DMSO-d_6

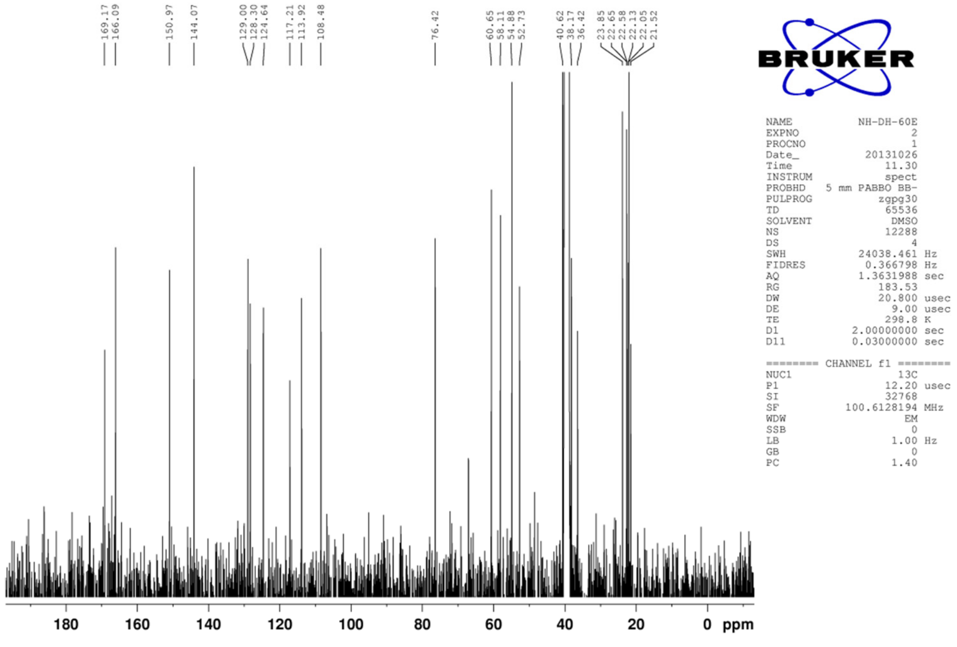

Figure S2. ^{13}C NMR spectrum of Compoud **1** in DMSO-d_6

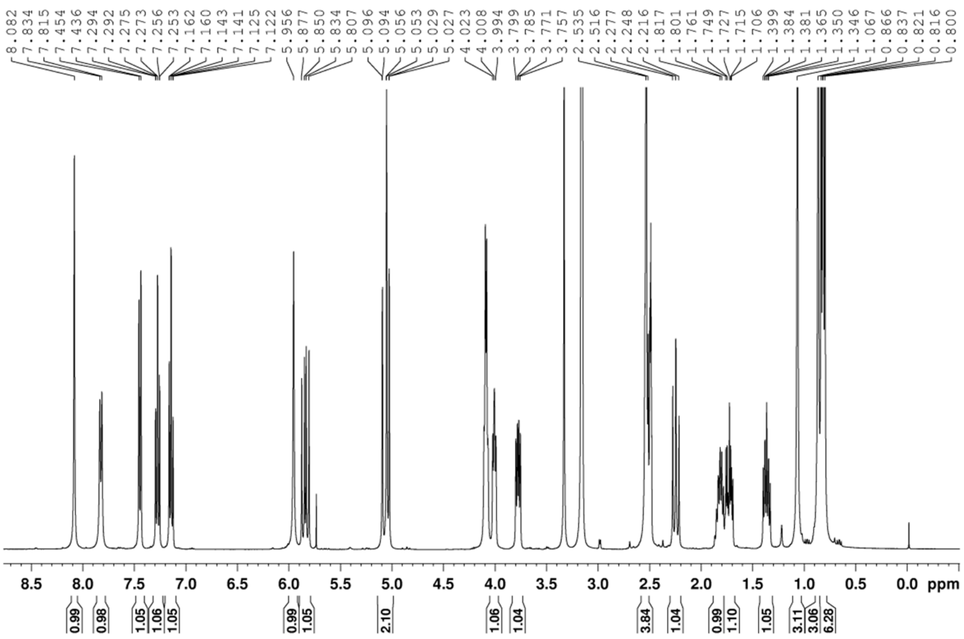

Figure S3. ^{1}H NMR spectrum of Compoud **2** in DMSO-d_6

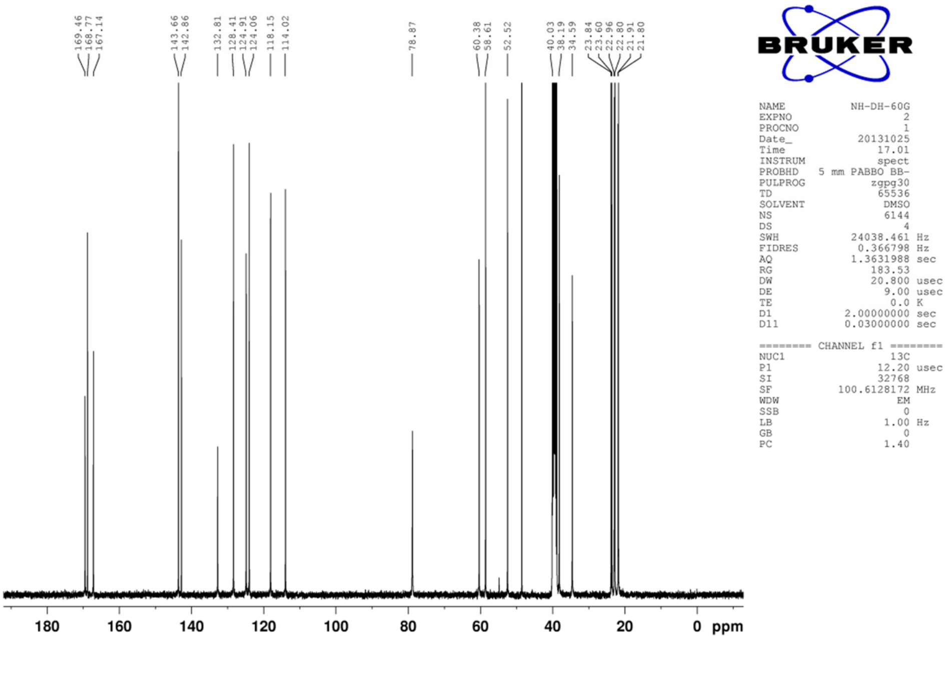

Figure S4. ^{13}C NMR spectrum of Compoud **2** in DMSO-d_6

Table S1. ^{1}H and ^{13}C NMR data of Compounds **1** and **2**.[a]

	**1**		**2**	
Position	δ_C	δ_H (J in Hz)	δ_C	δ_H (J in Hz)
1	NH	5.38, s	N	
2	76.4, CH	5.38, s	78.9, CH	5.95, br s
3	60.6, C		60.4, C	
4	124.6, CH	7.15, d (7.4)	124.9, CH	7.45, d (7.6)
5	117.2, CH	6.61, td (7.4, 1.0)	124.1, CH	7.14, td (7.6, 1.0)
6	128.3, CH	7.00, td (7.4, 1.0)	128.4, CH	7.27, td (7.6, 1.0)
7	108.5, CH	6.52, d (7.4)	118.1, CH	7.83, d (7.6)
8	151.0, C		142.8, C	
9	129.0, C		132.8, C	
10a	36.4, CH_2	2.32, dd (11.2, 6.8)	34.6, CH_2	2.51, m
10b		2.22, t (11.2)		2.24, t (11.2)
11	58.1, CH	4.00, t (5.2)	58.6, CH	4.09, t (5.9)
13	166.1, C		167.1, C	
14	52.7, CH	3.85, dd (10.0, 2.5)	52.5, CH	3.78, dd (10.0, 3.6)
15	NH	7.98, s	NH	8.08, s
16	169.2, C		168.8, C	
17a	38.2, CH_2	1.85, m	38.2, CH_2	1.81, m
17b		1.41, m		1.36, m
18	23.8, CH	1.74, m	23.8, CH	1.72, m
19	22.6, CH_3	0.82, d (6.5)	22.9, CH_3	0.82, d (6.5)
20	21.5, CH_3	0.82, d (6.5)	21.8, CH_3	0.82, d (6.5)
Isoprene	113.9, CH_2	5.05, dd (10.8, 1.2)	114.0, CH_2	5.05, d (10.8)
		5.05, dd (17.3, 1.2)		5.05, d (17.3)
	144.1, CH	5.98, dd (17.3, 10.8)	143.6, CH	5.83, dd (17.3, 10.8)
	40.6, C		40.0, C	
	22.1, CH_3	0.87, s	22.8, CH_3	0.86, s
	22.0, CH_3	1.03, s	21.9, CH_3	1.06, s
Acetyl			169.9, C	
			23.6, CH_3	2.54, s

[a] ^{1}H and ^{13}C NMR spectra of Compounds **1** and **2** were obtained at 400 and 100 MHz. All of these compounds were dissolved in DMSO-d_6.
